# 788 Upper Extremity and Lower Extremity Net Devices for Use after Application of Cultured Epidermal Autograft

**DOI:** 10.1093/jbcr/irac012.339

**Published:** 2022-03-23

**Authors:** Callie J Hayes, Walter R Anyan, Xavier Lucio, Daphne A Perry, Ronda Hopkins, Jillian S Clark, Lily Durose

**Affiliations:** University of Utah Burn Center, Salt Lake City, Utah; University of Utah Burn Center, Salt Lake City, Utah; University of Utah Burn Center, Salt Lake City, Utah; University of Utah Burn Center, Salt Lake City, Utah; University of Utah Burn Center, Salt Lake City, Utah; University of Utah Burn Center, Sandy, Utah; University of Utah Burn Center, Salt Lake City, Utah

## Abstract

**Introduction:**

Early positioning of the burn patient to prevent scar contracture is a fundamental aspect of the therapy treatment plan^1^. Positioning is complicated by different types of grafting including cultured epidermal autograft (CEA). Use of CEA requires a period of airing out and therefore positioning devices must allow adequate air flow to the posterior aspect of the grafted limb while immobilizing the extremity. Previous lower extremity net devices have been presented for use^2-3^, however this design increases hip flexion which can promote contracture. Additionally, there is no similar net device presented for use for the upper extremity.

**Methods:**

Both the upper extremity and lower extremity net device designs are made using 1” PVC pipe. A mix of Tee, 90⁰ and 45⁰ couplers are used to link together the PVC pipe. A PVC cutter is needed to cut the pieces. Once the pieces have been fit together and are aligned, the pieces are secured with a bonding PVC adhesive. The devices are then sanitized for patient use. A double layer of burn netting is used over the device to support the extremity while not allowing too much slack. The following graphics provide a basic template for an average adult adjusting lengths and width as needed. For the lower extremity, a measurement from the fold of the buttocks to the heel is needed to determine overall length of the device. The average cost for 1 lifter is $15-20. Additional custom plantar foot plates can be added to the lower extremity device to prevent ankle dorsiflexion as demonstrated by the poster presentation by Dean et. al. ^3^ For the upper extremity, an armboard or use of bedside table can be used to support the device. These particular designs are an outline for adult patients, although could be modified for pediatrics.

**Results:**

Net devices for the upper and lower extremity allow good positioning of limb while providing adequate air flow and off-loading during the required air out periods using CEA. These designs provide a template for burn therapists to utilize as a baseline. This particular lower extremity device decreases hip flexion as compared to other existing devices. The upper extremity template provides an option that has not previously been articulated.

**Conclusions:**

Positioning the upper and lower extremity after CEA application can be achieved using custom net devices. Net devices provide a cost-effective, quick and simple solution that allow adequate ventilation to the posterior aspect of the limb while maintaining a good position during immobilization.

